# HP-1γ Controls High-Affinity Antibody Response to T-Dependent Antigens

**DOI:** 10.3389/fimmu.2014.00271

**Published:** 2014-06-12

**Authors:** Ngoc Ha, Duc-Hung Pham, Aliakbar Shahsafaei, Chie Naruse, Masahide Asano, To-Ha Thai

**Affiliations:** ^1^Beth Israel Deaconess Medical Center, Department of Pathology, Harvard Medical School, Boston, MA, USA; ^2^Laboratory for Molecular Biodiscovery, Department of Pharmaceutical and Pharmacological Sciences, University of Leuven, Leuven, Belgium; ^3^Department of Pathology, Brigham and Women’s Hospital, Boston, MA, USA; ^4^Advanced Science Research Center, Kanazawa University, Kanazawa, Japan

**Keywords:** chromatin remodeling, epigenetics, adaptive immunity, germinal center response, CD8^+^ regulatory T cells

## Abstract

*In vitro* observations suggest a role for the mouse heterochromatin protein 1γ (HP-1γ) in the immune system. However, it has not been shown if and how HP-1γ contributes to immunity *in vivo*. Here we show that in mice, HP-1γ positively regulates the germinal center reaction and high-affinity antibody response to thymus (T)-dependent antigens by limiting the size of CD8^+^ regulatory T-cell (T_reg_) compartment without affecting progenitor B- or T-cell-development. Moreover, HP-1γ does not control cell proliferation or class switch recombination. Haploinsufficiency of *cbx-3* (gene encoding HP-1γ) is sufficient to expand the CD8^+^ T_reg_ population and impair the immune response in mice despite the presence of wild-type HP-1α and HP-1β. This is the first *in vivo* evidence demonstrating the non-redundant role of HP-1γ in immunity.

## Introduction

The adaptive immune system allows jawed vertebrates to distinguish self from non-self, to eliminate infectious agents, and to eradicate tumors. In addition, jawed vertebrates have the unique ability to store long-term immunological memory, thus enabling a rapid and vigorous adaptive immune response against previously encountered microbes. To achieve this outcome, diverse lymphocyte populations and their effector functions must be finely orchestrated and controlled. Dysregulation of any of these processes may result in the development of autoimmune diseases, inability to resolve infections, or failure to control the outgrowth of malignant cells. Therefore, the regulation of the adaptive immune response must occur on many levels, and there still remain novel genes and pathways yet to be uncovered.

The heterochromatin protein 1 (HP-1) family includes members that associate with modified histones, indicating that HP-1 proteins are involved in epigenetic modifications. HP-1 proteins are conserved from the yeast *Schizosaccharomyces pombe* (*S. pombe*) to mammals (1–5). The mammalian HP-1 family consists of three conserved members: HP-1α, HP-1β, and HP-1γ encoded by *cbx-5*, *cbx-1*, and *cbx-3*, respectively (2). Of particular significance to the immune system is the observation that HP-1γ is found associated with the transcription elongation complex containing RNA polymerase II (Pol II) within the coding region of the actively transcribed IL-2 gene in stimulated primary T cells (6). By contrast, during B-cell-development, HP-1γ associates with the silenced κ allele implicating HP-1γ in allelic exclusion (7). In addition, HP-1γ has been found associated with both heterochromatin and euchromatin suggesting that it participates in transcriptional repression and activation, respectively (4, 8, 9). HP-1γ interacts with the methyl groups of H3K9 through the chromodomain (CD) and with the methyl transferase SUV39-H1 and other proteins through the chromoshadow domain (CSD) (2, 3, 10). Despite these crucial *in vitro* observations, it is not understood if and how HP-1γ contributes to the regulation of immunity in mammals *in vivo*. Our interest in HP-1γ stems from efforts to identify novel targets of miR-155 (11). We find that HP-1γ expression is induced in activated mutant B cells suggesting that it might be an miR-155 target.

During a thymus (T)-dependent B-cell response, activated B cells migrate into follicles of secondary lymphoid organs. A fraction of activated B cells mediate a primary antibody (Ab) response through differentiation into plasma cells, others are recruited to the germinal center (GC) reaction (12, 13). In the GC, a specialized structure within the follicle, B cells undergo massive proliferation accompanied by class switch recombination (CSR) and somatic hypermutation (SHM) of rearranged immunoglobulin (Ig) V region genes. SHM leads to the acquisition of mutations that increase Ab affinity to the immunizing antigen (Ag), a process known as affinity maturation (12, 14, 15). The production of high-affinity, isotype-switched Ab is crucial for the clearance of many infectious pathogens and provides the basis for humoral immunity and vaccine efficacy.

Resident GC T follicular helper (T_FH_) cells make up a specialized subset of effector CD4^+^ T cells that are pivotal in affinity maturation by selecting activated B cells to enter the GC, regulating GC positive selection, and directing B-cell differentiation to plasma cells and memory B cells (16–18). Within the GC, T_FH_ cells develop in concert with GC B cells (19–24).

Early observations show that a subset of effector CD8^+^ T cells can suppress T-cell help to B cells (25). Recent studies demonstrate that these CD8^+^ regulatory T (T_reg_) cells control GC reaction and high-affinity Ab response to foreign T-dependent Ags as well as self-Ags by limiting the size of the T_FH_ compartment (26, 27). In mice, genetic disruption of the inhibitory interaction between CD8^+^ T_reg_ cells and their target Qa-1^+^ T_FH_ cells results in the development of systemic lupus erythematosus (SLE)-like autoimmune disease and the inability to mount a high-affinity Ab response to T-dependent Ags. These studies reveal the central role that CD8^+^ T_reg_ cells play in the control of the adaptive immune response. However, mechanisms that regulate the development and/or homeostasis of these cells remain elusive.

In this study, we uncover a novel molecular pathway that regulates the adaptive immune response to T-dependent Ags. We demonstrate that HP-1γ positively controls the GC reaction and high-affinity Ab response. HP-1γ does so by limiting the size of the CD8^+^ T_reg_ compartment. Haploinsufficiency of *cbx-3* results in the expansion of CD8^+^ T_reg_ cells and impaired immune response.

## Results

### B- or T-cell-development is not affected by HP-1γ deficiency

**Figure 1:**
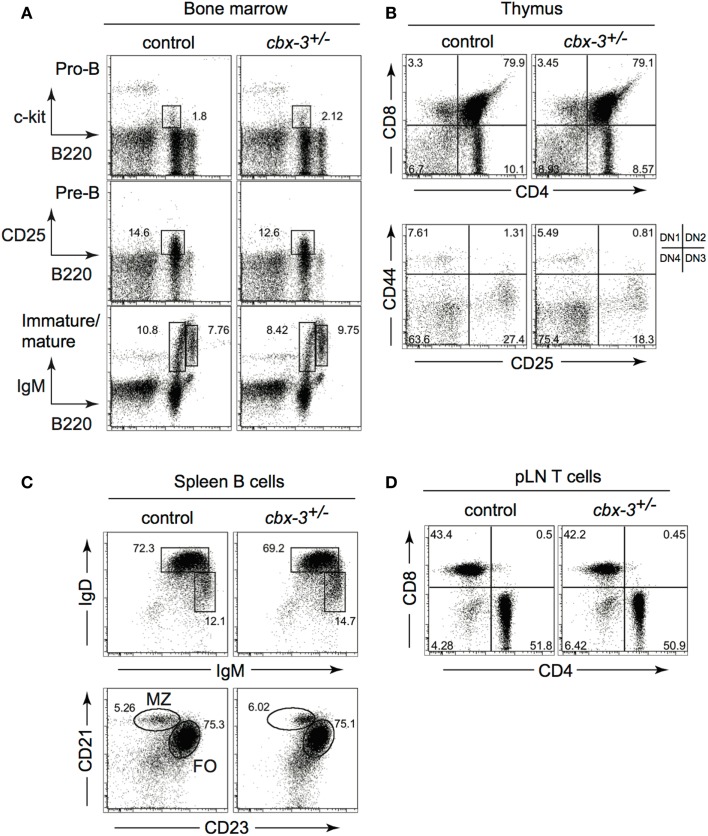
**B- or T-cell-development is not affected by HP-1γ deficiency**. **(A)** Bone marrow progenitor B-cell development from unimmunized wt littermate and *cbx-3*^+/−^ mice was determined by FACS. Progenitor B cells were gated on the lymphoid population. Pre-B cells were derived from the surface IgM (sIgM) negative lymphoid gate. **(B)** Thymi from unimmunized wt littermate and *cbx-3*^+/−^ mice were analyzed to assess progenitor T-cell-development. Upper plots were derived from the lymphoid gate; lower plots were gated on the CD4^−^CD8^−^ population. **(C)** All plots were gated on CD19^+^ lymphoid population. Frequency of mature (IgD^hi^IgM^lo^), marginal zone (CD21^hi^CD23^lo^), and follicular (CD21^lo^CD23^hi^) spleen B cells was determined by FACS. **(D)** Lymphoid cells from peripheral lymph nodes (pLNs) were gated on the CD3^+^ population, and analyzed to assess the development of mature CD4^+^ and CD8^+^ T cells. Results are representative of three independent experiments with *n* = 6 of each genotype.

Although *in vitro* studies suggest a role for HP-1γ in the immune system, it has not been determined if it contributes to immunity *in vivo*. The *cbx-3* (gene encoding HP-1γ) mutant mouse was generated by gene-trapping technology as described previously (10, 28). We found that *cbx-3^−^*^/−^ mice died perinatally. Because haploinsufficiency of genes involved in epigenetic modifications has been shown to alter cellular functions (29), we asked if haploinsufficiency of *cbx-3* was sufficient to affect the immune system. First we assessed if *cbx-3* deficiency influenced progenitor lymphoid development. A survey of the bone marrow (BM) and thymus showed that progenitor B and T cells developed normally in *cbx-3*^+/−^ mice compared to littermate controls (Figures 1A,B). Mature B- and T-cell-development also remained normal in *cbx-3*^+/−^ mice (Figures 1C,D). Thus, HP-1γ is not required for progenitor or mature B- and T-cell-development.

### HP-1γ deficiency results in impaired germinal center reaction

**Figure 2:**
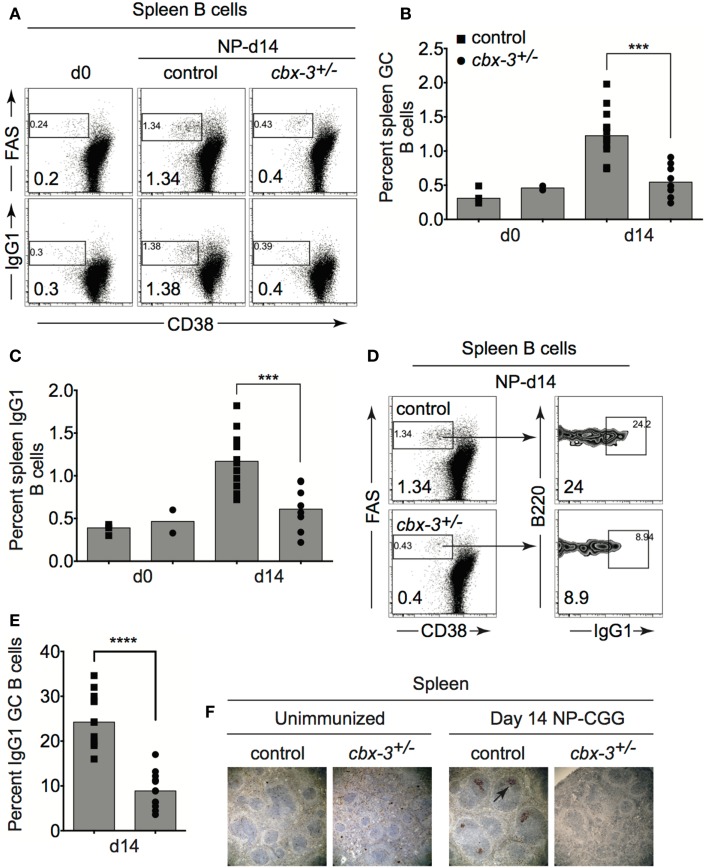
**Heterochromatin protein 1γ deficiency results in impaired germinal center reaction**. **(A)** Wt littermate and *cbx-3*^+/−^ mice were immunized with NP-CGG in alum. On day 14 after immunization, mice were analyzed by FACS to determine the frequency of spleen CD38^lo/−^FAS^+^ GC and switched CD38^lo∕−^${\text{IgG}}_{1}^{+}$ B cells from the B220^+^ gate. Numbers in left bottom corners indicate percent cells. **(B)** Plot depicts the compilation of GC B-cell frequency from experiments in **(A)**. Each symbol represents an individual mouse. Bars represent median ***p = 0.0002. **(C)** Plot summarizes switched IgG_1_ B-cell frequency from experiments in **(A)**. Bars represented median ***p = 0.0007. Each symbol represents an individual mouse. **(D)** Frequency of ${\text{IgG}}_{1}^{+}$ GC B cells was determined from **(A)**, gated on B220^+^CD38^lo/−^FAS^+^ GC B cells. Numbers in left bottom corners indicate percent cells. **(E)** Plot summarizes the percent ${\text{IgG}}_{1}^{+}$ GC B cells from **(D)**. Bars represent median, ****p < 0.0001. Each symbol represents an individual mouse. **(F)** Immunohistochemistry of spleen sections from unimmunized and day 14 NP-immunized mice were stained for PNA (brown) to detect GCs (arrow). Spleens were from mice in **(A)**. Images are shown at 100× magnification. Statistical analysis was performed with GraphPad one-way ANOVA. *N* = 8–12 for each genotype.

To determine the physiological function of HP-1γ in the adaptive immune response in mice, we immunized littermate control and *cbx-3*^+/−^ mice with the T-dependent Ag 4-hydroxy-3-nitrophenylacetyl hapten conjugated to chicken gamma globulin (NP-CGG) in alum. On day 14 after immunization, the expected frequency of spleen B220^+^CD38^lo/^*^−^*FAS^+^ GC as well as switched B220^+^CD38^lo∕−^${\text{IgG}}_{1}^{+}$ B cells was obtained from littermate controls, demonstrating that the GC reaction occurred normally (Figure 2). In contrast, the GC response was impaired in *cbx-3*^+/−^ mice. On day 14, the percent of spleen GC B cells in *cbx-3*^+/−^ mice decreased by 2.3-fold compared to control mice (*p* = 0.0002, Figures 2A,B,F). Correspondingly, there was a twofold reduction in the percent of switched ${\text{IgG}}_{1}^{+}$ spleen B cells in *cbx-3*^+/−^ mice compared to control mice (*p* = 0.0007, Figures 2A,C). Similarly, the frequency of ${\text{IgG}}_{1}^{+}$ GC B cells in *cbx-3*^+/−^ mice was reduced by 2.8-fold compared to wild-type littermate mice (Figures 2D,E, *p* < 0.0001). The spleen architecture of wt littermate and mutant mice remained intact; and more peanut agglutinin (PNA) positive GCs were detected in wt littermate mice than in mutant mice on day 14 after immunization with NP (Figure 2F). These results demonstrate that HP-1γ positively regulates the GC reaction and production of ${\text{IgG}}_{1}^{+}$ B cells, and haploinsufficiency of *cbx-3* is sufficient to impair these processes. The defect cannot be compensated for by the presence of wild-type HP-1α and HP-1β in *cbx-3*^+/−^ mice suggesting that HP-1γ has a non-redundant function in immunity *in vivo*.

### *Cbx-3*^+/−^ mice fail to mount high-affinity NP antibody response

**Figure 3:**
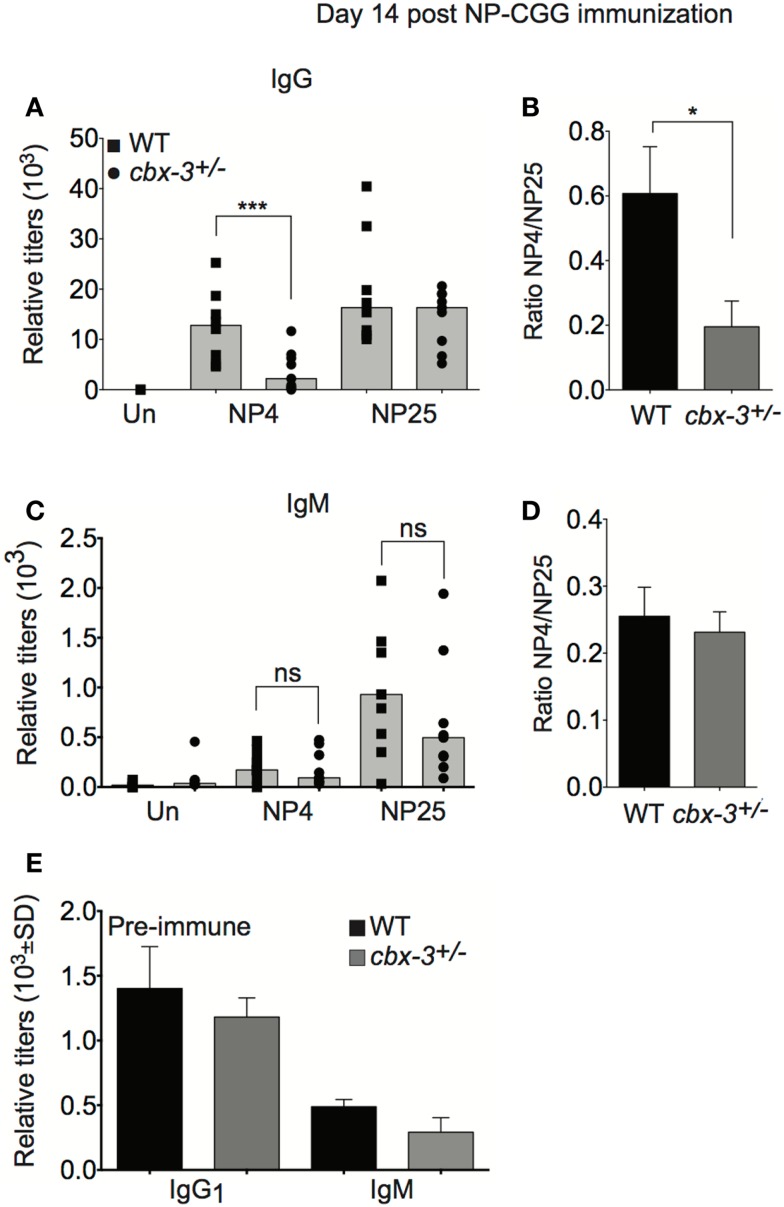
***Cbx-3*^+/−^ mice fail to mount the high-affinity NP antibody response**. **(A)** ELISA analysis was carried out to measure high-affinity IgG_1_ anti-NP4-BSA and total IgG_1_ anti-NP25-BSA antibody activity in sera from mice immunized with NP-CGG in alum. Each symbol represents an individual mouse. Bars represent median. Statistical analysis was performed with GraphPad one-way ANOVA. Day 14, *n* = 9 for each genotype, ***p = 0.006. **(B)** High-affinity serum IgG_1_ anti-NP Ab activity was determined by calculating the ratio of anti-NP4/NP25, *p = 0.02. **(C)** Serum IgM anti-NP4 and anti-NP25 antibody activity was analyzed by ELISA to NP-BSA as in **(A)**. **(D)** Plot depicts ratio of IgM anti-NP4 over anti-NP25. **(E)** Total serum IgG_1_ and IgM titers of pre-immune sera from **(A)** were measured by ELISA.

To determine if the diminished GC reaction in *cbx-3*^+/−^ mice results in defective serum anti-NP Ab response, we measured anti-NP activity in sera obtained from *cbx-3*^+/−^ and wt littermate mice on day 14 after NP immunization. Serum high- and low-affinity responses to NP can be measured by enzyme-linked immunosorbent assay (ELISA) using NP4 and NP25 Ags, respectively. On day 14 after immunization, serum IgG anti-NP25 Ab activity increased in littermate control and mutant mice compared to unimmunized animals, and the level was similar between the two groups. By contrast, the amount of serum anti-NP4 antibodies in littermate control mice was 4.75-fold higher than *cbx-3*^+/−^ mice (*p* = 0.006, Figure 3A). Accordingly, the ratio of NP4/NP25 Ab titer was threefold lower in *cbx-3*^+/−^ mice compared to littermate control mice (*p* = 0.02, Figure 3B). The low-affinity response was not affected by *cbx-3* haploinsufficiency. Both littermate control and mutant mice produced low amounts of serum IgM Abs against NP, and the majority of IgM antibodies were of low-affinity (Figures 3C,D). There was no difference in the production of total pre-immune serum IgG_1_ and IgM between wt littermate control and mutant mice (Figure 3E). Thus *cbx-3*^+/−^ mice could not mount high-affinity Ab response to NP.

### HP-1γ does not regulate B-cell proliferation or class switch recombination

**Figure 4:**
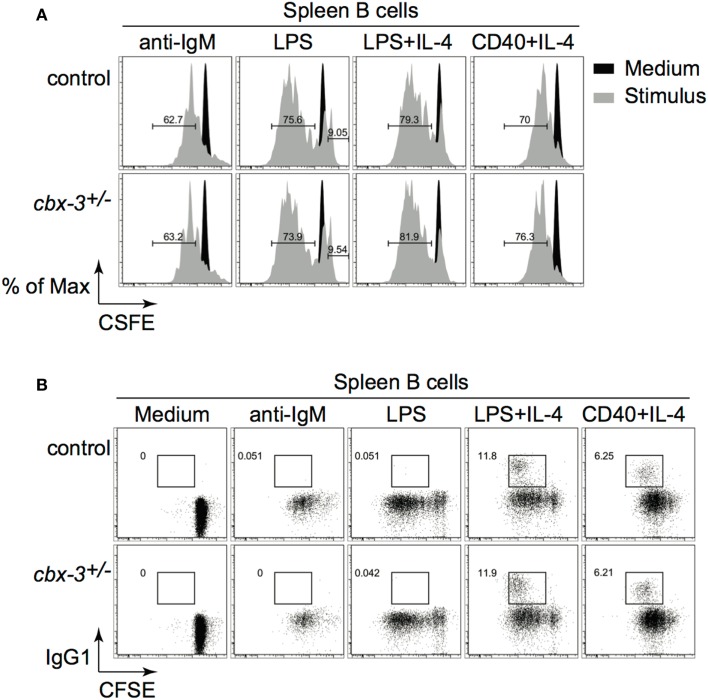
**Heterochromatin protein 1γ does not regulate B-cell proliferation or class switch recombination**. **(A)** Naïve spleen B cells were labeled with CFSE and stimulated with various stimuli for 3 days. Cell division was determined using the FlowJo Proliferation Platform software. **(B)** IgG_1_ switching was determined from the same cultures as in **(A)**. Results are representative of three independent experiments with six mice per genotype. Analysis was performed on cells derived from the live gate.

To rule out the possibility that reduced GC and Ab responses resulted from defects in proliferation or class switching after Ag encounter, we carried out *in vitro* proliferation/switch assays. Spleen B cells from *cbx-3*^+/−^ mice proliferated and switched as well as littermate control B cells when activated through the B-cell receptor, Toll-like receptor (TLR) 4, or CD40 plus IL-4 (Figures 4A,B). Therefore, HP-1γ deficiency results specifically in impaired high-affinity, not total NP Ab response. HP-1γ does not control the IgM response to NP. Because HP-1γ deficiency does not perturb proliferation or switching, the defect in high-affinity Ab response observed in *cbx-3*^+/−^ mice implies that HP-1γ may regulate Ab affinity maturation.

### The T follicular helper cell population is reduced in *Cbx-3*^+/−^ mice

**Figure 5:**
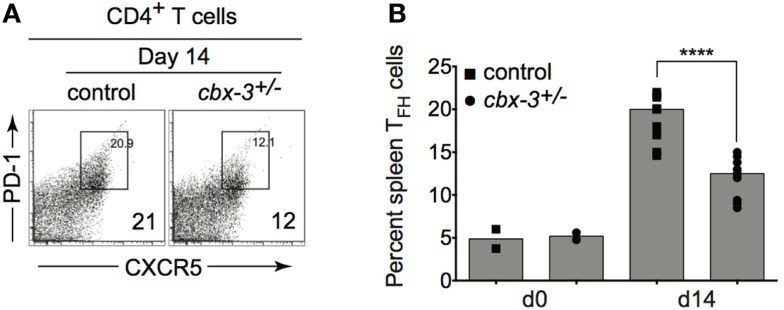
**The T follicular helper cell copulation is reduced in *cbx-3*^+/−^ mice**. **(A)** The frequency of spleen TCRβ^+^CD4^+^CXCR5^hi^PD-1^hi^ T_FH_ population was determined by FACS analysis. Numbers in right bottom corners indicate the percent cells. CXCR5^hi^PD-1^hi^ T_FH_ cells were gated on the TCRβ^+^CD4^+^ population. **(B)** Plot represents a compilation of the percent of T_FH_ population from **(A)**. Each symbol denotes an individual mouse. Bars represent median. Statistical analysis was performed with GraphPad one-way ANOVA. Day 14, *n* = 8–12 for each genotype, ****p < 0.0001.

T follicular helper cells play a crucial role in affinity maturation in part by selecting B cells to enter the GC, regulating GC positive selection, and directing B-cell differentiation to plasma cells and memory B cells. Hence, the high-affinity Ab response defect seen in *cbx-3*^+/−^ mice may arise from inefficient T_FH_ support. Fluorescence-activated cell sorting (FACS) analysis showed that as the immune response proceeded to day 14, the frequency of TCRβ^+^CD4^+^CXCR5^hi^PD-1^hi^ T_FH_ cells decreased by 1.7-fold in *cbx-3*^+/−^ mice compared to wt littermate control mice (*p* < 0.0001, Figures 5A,B). Moreover, we did not detect any differences in *Bcl6*, *Prdm1*, or *Aicda* expression between wt littermate control and mutant mice suggesting that GC and plasma cell differentiation was not affected by HP-1γ deficiency (data not shown). Thus, HP-1γ governs Ab affinity maturation perhaps by controlling the size of the T_FH_-cell compartment during an immune response to T-dependent Ags.

### The GC reaction defect in *Cbx-3*^+/−^ mice is not intrinsic to B or T_FH_ cells

**Figure 6:**
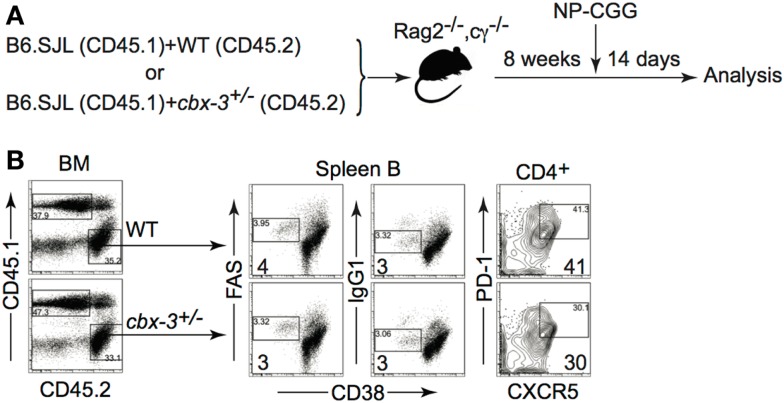
**The GC reaction defect in *Cbx-3*^+/−^ mice is not intrinsic to B or T_FH_ cells**. **(A)** *cbx-3*^+/+^*/cbx-3*^+/−^ mixed bone marrow chimeras were generated in B6-*Rag2*^−^*^/^*^−^*cγ*^−^*^/^*^−^recipients at a 1:1 ratio of either CD45.1 *cbx-3*^+/+^/CD45.2 *cbx-3*^+/+^ or CD45.1 *cbx-3*^+/+^/CD45.2 *cbx-3*^+/−^ bone marrow. Eight weeks after reconstitution chimeric mice were immunized with NP-CGG in alum. **(B)** On day 14, spleens were analyzed to determine the frequency of GC B cells, switched ${\text{IgG}}_{1}^{+}$ B cells and T_FH_ cells derived from CD45.2 (control *cbx-3*^+/+^ or *cbx-3*^+/−^) donor bone marrow in each mouse. Numbers in bottom left corners of FACS plots indicate percent GC B cells. Numbers in bottom right corners denote percent T_FH_ cells. CD45.1 and CD45.2 populations were derived from the lymphoid gate. GC and ${\text{IgG}}_{1}^{+}$ B cells were derived from the B220^+^ gate. CXCR5^hi^PD-1^hi^ T_FH_ cells were gated on the TCRβ^+^CD4^+^ population.

To determine if T_FH_ cells were directly responsible for the GC phenotype observed, we generated *cbx-3*^+/+^*/cbx-3*^+/−^ mixed BM chimeras. Recombinase activating gene 2 and common γ chain double knock out (B6-*Rag2^−/−^cγ^−/−^*) mice were reconstituted with a 1:1 mix of either CD45.1 *cbx-3*^+/+^/CD45.2 *cbx-3*^+/+^ or CD45.1 *cbx-3*^+/+^/CD45.2 *cbx-3*^+/−^ BM. Eight weeks after reconstitution chimeric mice were immunized with NP-CGG in alum. On day 14 after immunization, mice were analyzed to determine the frequency of GC B cells, switched ${\text{IgG}}_{1}^{+}$ B cells and T_FH_ cells derived from CD45.2 (control *cbx-3*^+/+^ or *cbx-3*^+/−^) donor BM in each mouse (Figure 6A). FACS analysis showed that CD45.2 *cbx-3*^+/+^ and CD45.2 *cbx-3*^+/−^ chimeric mice had similar percentage of GC and ${\text{IgG}}_{1}^{+}$ B cells as well as T_FH_ cells (Figure 6B). Therefore, the GC defect observed in *cbx-3*^+/−^ is not intrinsic to B or T_FH_ cells.

### CD122^+^Ly49^+^CD3^+^CD8^+^ regulatory T-cell compartment is expanded in *Cbx-3*^+/−^ mice

**Figure 7:**
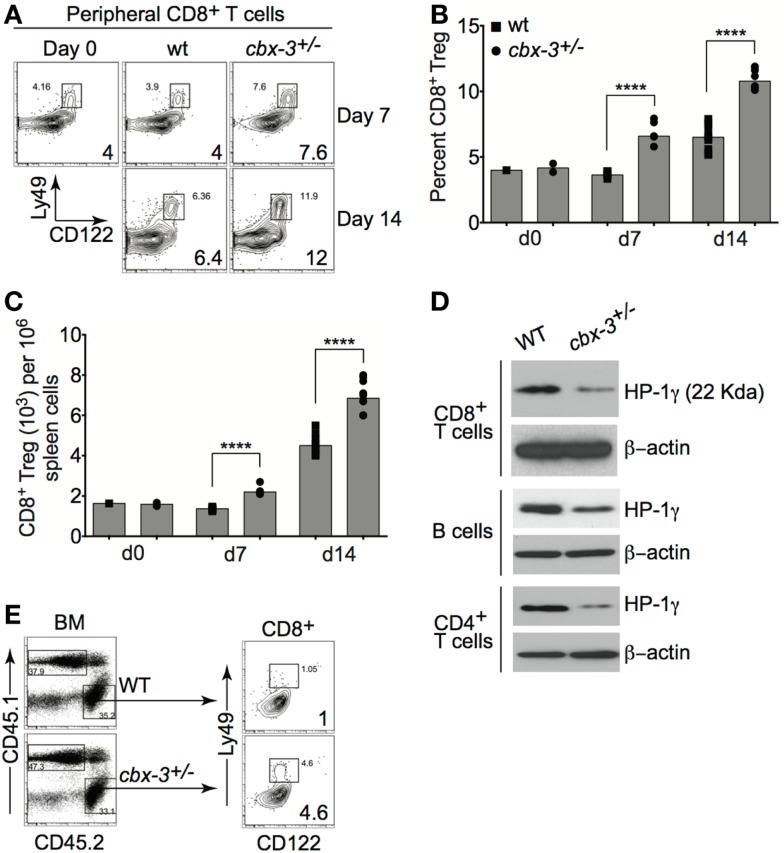
**CD122^+^Ly49^+^CD3^+^CD8^+^ regulatory T-cell compartment is expanded in *cbx-3*^+/−^ mice**. **(A)** On days 7 and 14 after immunization, percent of spleen CD122^+^Ly49^+^CD3^+^CD8^+^ regulatory T-cell (T_reg_) population from wt littermate and *cbx-3*^+/−^ mice was assessed by FACS. Numbers in lower right corners indicate percent cells. CD122^+^Ly49^+^CD8^+^ T_reg_ cells were gated on the CD3^+^ population. **(B,C)** Plots represent a compilation of the percent and number of CD8^+^ T_reg_ population from **(A)**. Each symbol denotes an individual mouse. Bars represent median. Statistical analysis was performed with GraphPad one-way ANOVA. Day 7, *n* = 5 for each genotype; ****p < 0.0001. Day 14, *n* = 8–12 for each genotype; ****p < 0.0001. **(D)** Purified CD8^+^CD44^−^, CD4^+^CD25^−^, and CD43^−^ B cells were collected from spleen and peripheral lymph nodes. Blots were probed with anti-total HP-1γ (22 kDa) and anti-β-actin (42 kDa). Results are representative of two independent experiments; *n* = 4 mice of each genotype. **(E)** *cbx-3*^+/+^*/cbx-3*^+/−^ mixed bone marrow chimeras were generated as in Figure 6A. Percent CD8^+^ T_reg_ cells were determined by FACS. Numbers in lower right corners indicate percent cells. CD45.1 and CD45.2 populations were derived from the lymphoid gate. CD122^+^Ly49^+^CD8^+^ T_reg_ cells were gated on the CD3^+^ population.

Recently, Kim and colleagues showed that CD122^+^Ly49^+^CD3^+^ CD8^+^ regulatory T (T_reg_) cells served to inhibit the expansion of T_FH_ population during an immune response to foreign Ags as well as to self-Ags (26, 27). Thus it is plausible that reduction in the T_FH_ compartment in *cbx-3*^+/−^ mice may be due to an increase in CD8^+^ T_reg_ cells within the CD8^+^ T-cell compartment. On days 7 and 14 after immunization, compared to wt littermate mice, *cbx-3*^+/−^ mice had 2.3- and 1.8-fold higher frequency of spleen CD8^+^ T_reg_ cells, respectively (*p* < 0.0001, Figures 7A,B). Correspondingly, the number of spleen CD8^+^ T_reg_ cells in *cbx-3*^+/−^ mice increased by 1.8- and 1.75-fold on days 7 and 14, respectively (*p* < 0.0001, Figure 7C). Next, western blots were carried out to assess the expression status of HP-1γ in mutant CD8^+^ T cells. To our surprise, CD8^+^ and CD4^+^ T cells as well as B cells from *cbx-3*^+/−^ mice expressed much less HP-1γ than control cells despite the presence of one wild-type allele (Figure 7D). To ensure that the expansion of CD8^+^ T_reg_ cells in *cbx-3*^+/−^ mice was intrinsic to the CD8^+^ T-cell population, mixed BM chimeras were generated as described in Figure 6A. Mice were allowed to reconstitute for 8 weeks. On day 14 after immunization, reconstituted mice were analyzed to assess the frequency of T_reg_ cells derived from CD45.2 (control *cbx-3*^+/+^ or *cbx-3*^+/−^) donor BM in each mouse. As shown in Figure 7E, CD45.2 *cbx-3*^+/−^ chimeric mice had 4.6-fold more CD8^+^ T_reg_ cells than CD45.2 *cbx-3*^+/+^ control mice. Thus, the CD8^+^ T_reg_ population expanded in *cbx-3*^+/−^ mice. These results suggest that HP-1γ limits the size of CD8^+^ T_reg_ cells during an immune response, and the effects are intrinsic to these cells.

### *Cbx-3*^+/−^ CD8^+^ regulatory T cells directly control T-dependent immune response

**Figure 8:**
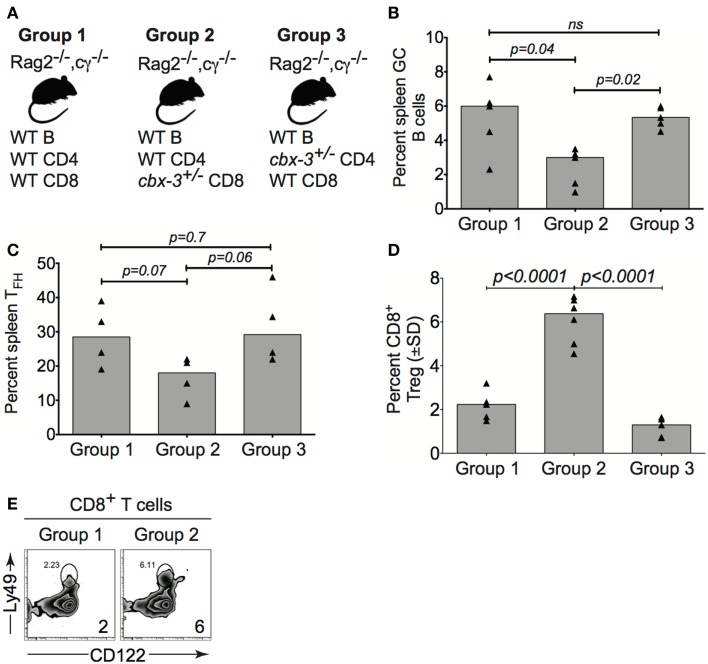
***Cbx-3*^+/−^ CD8^+^ T cells directly control the T-dependent immune response**. **(A)** Purified wt or *cbx-3*^+/−^ spleen and pLN lymphocytes were adoptively transferred into B6-*Rag2*^−^*^/^*^−^*cγ*^−^*^/^*^−^recipients: group 1 (control, *n* = 5) received wt B cells, CD4^+^ and CD8^+^ T cells; group 2 (experimental, *n* = 5) received B cells and CD4^+^ T cells from wt littermate mice and CD8^+^ T cells from *cbx-3*^+/−^ mice; group 3 (control, *n* = 5) received B cells and CD8^+^ T cells from wt littermate mice and CD4^+^ T cells from *cbx-3*^+/−^ mice. **(B,C)** On day 10 after NP-CGG in alum immunization, spleens of recipients were analyzed by FACS to determine the frequency of GC (from the B220^+^gate) and T_FH_ (from the TCRβ^+^CD4^+^ gate) populations. **(D,E)** The frequency of CD122^+^Ly49^+^CD8^+^ T_reg_ cells (from the CD3^+^ gate) was determined by FACS **(E)** and plotted **(D)**. Numbers on bottom right corners indicate percent cells. Statistical analysis was performed with GraphPad one-way ANOVA; ns, not significant.

To investigate if CD8^+^ T_reg_ cells from *cbx-3*^+/−^ mice directly controlled the Ab response, adoptive transfers into B6-*Rag2^−/−^cγ^−/−^*recipients were performed. Group 1 (control) recipients received B cells, CD4^+^ and CD8^+^ T cells from wt littermate mice; group 2 (experimental) received B cells and CD4^+^ T cells from wt littermate mice, and CD8^+^ T cells from *cbx-3*^+/−^ mice; group 3 (control) received B cells and CD8^+^ T cells from wt littermate mice, and CD4^+^ T cells from *cbx-3*^+/−^ mice (Figure 8A). On day 10 after immunization with NP-CGG in alum, recipients were analyzed to assess the status of the GC response. The percent of GC B cells from group 2 was 2.2-fold lower than groups 1 and 3 (*p* = 0.04 and *p* = 0.02, respectively, Figure 8B). Additionally, group 2 recipients had 1.7- and 1.9-fold less T_FH_ cells than groups 1 and 3, respectively (Figure 8C). By contrast, the frequency of CD8^+^ T_reg_ cells from group 2 was three- and fourfold higher than groups 1 and 3, respectively (Figures 8D,E). Thus, HP-1γ-deficient CD8^+^ T cells alone have the propensity to reduce the GC response. The results suggest that HP-1γ positively regulates GC and high-affinity Ab responses to T-dependent Ags by curtailing the ability of CD8^+^ T cells to inhibit an immune response.

## Discussion

The regulation of the adaptive immune response is multilayered, requiring the participation of multiple cells and their proper functions. Here we uncover a novel function for the chromatin-remodeling factor HP-1γ in governing immunity.

*Cbx-3* was cloned nearly two decades ago and yet very little is known of its physiological function in the mammalian immune system (30). Our results reveal an essential role for HP-1γ in the control of the adaptive immune response in mice. We demonstrate that HP-1γ has a positive impact on the GC reaction and high-affinity Ab response to T-dependent Ags. Mainly, *cbx-3*^+/−^ mice fail to mount an effective GC reaction and high-affinity IgG_1_ Ab response, whereas the low-affinity IgG_1_ Ab response remains intact. The GC reaction and high-affinity response defects are accompanied by a reduction in the T_FH_ compartment. The fact that neither low-affinity IgG_1_ Ab nor IgM response is affected in *cbx-3*^+/−^ mice indicates that HP-1γ may not be essential for extrafollicular reaction. The presence of wild-type HP-1α and HP-1β proteins cannot override defects in GC reaction and high-affinity Ab response seen in *cbx-3*^+/−^ mice suggests that HP-1γ has a non-redundant regulatory function in immune response to T-dependent Ags. The function of HP-1γ in immune response is not intrinsic to B or T_FH_ cells.

*In vitro* observations suggest that HP-1γ associates with the silenced κ allele thus may be involved in light chain allelic exclusion during B-cell-development (7). Our results demonstrate that light chain allelic exclusion and B-cell-development in the BM occur normally in *cbx-3*^+/−^ mice. However, our data do not rule out the possibility that other HP-1 proteins, HP-1α and HP-1β, may compensate for HP-1γ deficiency during B-cell-development.

Recent studies have shown that a subpopulation of effector CD8^+^ T cells, known as CD8^+^ T_reg_ cells, control GC reaction and high-affinity Ab response to foreign T-dependent Ags as well as self-Ags by limiting the size of the T_FH_ compartment (27). However, mechanisms that regulate the development or homeostasis of these cells remain elusive. Here, we reveal a novel molecular pathway that controls CD8^+^ T_reg_ cells in mice after immunization. We show that, through its non-redundant function, HP-1γ limits the size of the CD8^+^ T_reg_ population thus allowing the immune response to foreign T-dependent Ags to proceed. In mice, HP-1γ deficiency results in the expansion of these cells and reduction of T_FH_ population, which leads to the abrogation of GC reaction and high-affinity Ab response. The level of HP-1γ present in mutant cells is much less than expected despite the presence of one wild-type allele, implying that HP-1γ may also regulate its own expression. HP-1γ deficiency only affects CD8^+^ T-cell function despite the fact that mutant CD4^+^ and B cells also express low amounts of HP-1γ, suggesting that in these cells HP-1γ may regulate the expression of genes that are not essential to NP-response.

It remains to be determined how HP-1γ controls the development/homeostasis of CD8^+^ T_reg_ cells, and if HP-1γ deficiency would alleviate autoimmunity. We speculate that HP-1γ may control the expression and/or function of a transcription factor(s), which governs CD8^+^ T_reg_ development/homeostasis. HP-1γ does so perhaps by maintaining a chromatin conformation that is unfavorable to the expression and/or function of this putative transcription factor(s). Future genome wide experiments will allow us to map the changing epigenomic landscape in HP-1γ sufficient and deficient CD8^+^ T cells. These ongoing studies will expand our understanding of mechanisms by which HP-1γ, through its ability to remodel the chromatin, regulates immunity.

In summary, this study shows for the first time that in mice the non-redundant regulatory function of HP-1γ governs GC and high-affinity Ab responses by limiting the pool of CD8^+^ T_reg_ cells.

## Materials and Methods

### Mice

*cbx-3* mutant mice were generated, as described in Ref. (10, 28). Mice were backcrossed to C57BL/6 for 12 generations. B6-*Rag2^−/−^C*γ*^−/−^* and B6.SJL mice were purchased from Taconic. All mice were maintained in specific pathogen-free conditions. All mouse protocols were approved by the BIDMC Institutional Animal Care and Use Committee.

### Fluorescence-activated cell sorting

Fluorescence-activated cell sorting was performed on the BD 5-laser LSR II. Analysis was carried with FlowJo software (Tree Star, Inc.). All fluorochrome-conjugated antibodies were purchased from Biolegend or BD Biosciences. The following antibodies were used: ckit-APC (1:200); CD25-PE (1:200); IgM-FITC (1:500); CD8-Pacific blue (1:200); CD8-APC-Cy7 (1:300); CD8-PE-Cy7 (1:200); Ly-49-FITC (1:100); CD44-Pacific blue (1:200); IgD-PE (1:500); CD21-APC (1:200); CD23-PE (1:150); CD19-PE-Cy7 (1:300); B220-Pacific blue (1:300); CD38-APC (1:200); IgG1-FITC (1:50); FAS-PE (1:200); CD4-FITC (1:200); CD4-PE (1:150); TCRβ-Brilliant-Violet 412 (1:200); PD1-PE-Cy7 (1:100); CXCR5-Biotin (1:100); SA-PerCP (1:100); CD45.1-FITC (1:150); CD45.2-PE-Cy7 (1:100); CD45.2-Pacific blue (1:200); CD3-APC (1:200); CD122-Pacific blue (1:200).

### T-dependent immune response

Adult mice (7–8-week-old) were immunized with 50 μg of the T-dependent Ag 4-hydroxy-3-nitrophenylacetyl hapten conjugated to chicken gamma globulin (NP-CGG, BioSearch Technologies) per mouse in alum (Thermo Scientific) (ratio 1:1). Immune sera were obtained at days 7 and 14 after immunization. FACS analysis was performed on the same days.

### Immunohistochemistry

Immunohistochemistry was performed using 4 μm thick formalin-fixed, paraffin-embedded tissue sections. Briefly, slides were soaked in xylene, passed through graded alcohols, and put in distilled water. Slides were then pre-treated with 1.0-mM EDTA, pH 8.0, or 1.0 mM citrate (Zymed) in a steam pressure cooker (Decloaking Chamber, BioCare Medical) as per manufacturer’s instructions, followed by washing in distilled water. All subsequent steps were performed at room temperature in a hydrated chamber. Slides were pre-treated with Peroxidase Block (DAKO) for 5 min to quench endogenous peroxidase activity, followed by Serum free Protein Block (DAKO) for 20 min. Biotinylated PNA (Vector Laboratories) was applied for 1 h (all diluted in DAKO diluents). Slides were washed in 50 mM Tris–Cl, pH 7.4. Slides were washed again, and detected with anti-streptavidin–HRP Envision + kit (DAKO) as per manufacturer’s instructions. After further washing, immunoperoxidase staining was developed using a DAB chromogen (DAKO) and counterstained with hematoxylin. Images were acquired with the Nikon Eclipse E600 and SPOT Insight four camera and software.

### Enzyme-linked immunosorbent assay

Antibody response to NP was determined by ELISA using NP(4)-BSA or NP(25)-BSA (BioSearch Technologies) from days 7 and 14 immune sera. ELISA was performed as described (11).

### *In vitro* B-cell activation and isotype switch assay

MACS-purified (Miltenyi Biotec) CD43*^−^* or CD19^+^ B cells were activated *in vitro* at a density of 1–3 × 10^6^ cells/ml with 2 μg/ml of anti-CD40 clone HM40-3 (eBiosciences) plus 25 ng/ml of recombinant mouse IL-4 (R&D Systems), 10 μg/ml of goat F(ab′)_2_ anti-mouse IgM (Jackson Immunoresearch), LPS (20 μg/ml), or LPS + IL-4 (Sigma).

### Proliferation assays and analysis

MACS-purified CD43*^−^* B cells labeled with CFSE were activated with indicated stimuli as above for 3 or 4 days. Data were analyzed using the proliferation platform of the FlowJo software (Tree Star Inc.).

### Generation of bone marrow chimeras

Bone marrow cells from femurs and tibias of 4-week-old B6.SJL mice (CD45.1) were mixed with either 4-week-old *cbx3*^+/−^ or littermate control mice (CD45.2) at a 1:1 ratio. Mixed BM cells (2 × 10^6^) were injected i.v. into 7-week-old *Rag2^−/−^C*γ*^−/−^*recipients. Eight weeks after BM reconstitution, recipients were immunized with NP-CGG in alum (ratio 1:1). Analysis was carried out 14 days after immunization.

### Western blots

Purified CD8^+^CD44^−^ (1 × 10^6^) cells were lysed with radio-immunoprecipitation assay (RIPA) buffer (Boston BioProducts) containing protease inhibitor cocktail (Roche) on ice for 30 min. Cells were centrifuged at 14,000 rpm for 15 min at 4°C. Protein concentration was determined by Bio-Rad Protein Assay Kit (Bio-Rad). Ten micrograms of protein extracts were denatured at 95°C for 10 min, separated by SDS-PAGE, and transferred onto PVDF membranes (EMD Millipore). Membranes were probed with antibodies against HP-1γ (Cell Signaling Technology) or β-actin (Sigma Aldrich). Proteins of interest were detected with HRP-conjugated secondary antibodies and visualized with the Pierce ECL Western blotting substrate (Thermo Scientific).

### Adoptive transfer

B, CD8^+^, and CD4^+^ cells were prepared from spleen and lymph nodes of 7-week-old *cbx3*^+/−^ and wt littermate mice as described (18). 2 × 10^6^ wild-type B cells, 1 × 10^6^ wild-type or mutant CD8^+^, and 1 × 10^6^ wild-type or mutant CD4^+^ cells were injected i.v. into 7-week-old *Rag*^−^*^/^*^−^*C*γ^−^*^/^*^−^recipients. The following day, recipients were immunized with NP-CGG in alum (ratio 1:1). Analysis was carried out 10 days after immunization.

### Statistical and graph analysis

*P* values were calculated using one-way ANOVA and graphs were plotted with Prism 6 (GraphPad Software).

## Author Contributions

Ngoc Ha, Duc-Hung Pham, and Aliakbar Shahsafaei carried out all experiments; Chie Naruse and Masahide Asano generated the *cbx-3* mutant mice and provided advice on their use; To-Ha Thai conceived and directed all research, and along with Ngoc Ha and Duc-Hung Pham prepared the manuscript.

## Conflict of Interest Statement

The authors declare that the research was conducted in the absence of any commercial or financial relationships that could be construed as a potential conflict of interest.
